# Behind the Optimization of the Sensor Film: Bioconjugation of Triangular Gold Nanoparticles with Hemoproteins for Sensitivity Enhancement of Enzymatic Biosensors

**DOI:** 10.3390/bios13040467

**Published:** 2023-04-10

**Authors:** Miriam Chávez, Ángela Fernandez-Merino, Rafael del Caño, Guadalupe Sánchez-Obrero, Rafael Madueño, Manuel Blázquez, Teresa Pineda

**Affiliations:** Department of Physical Chemistry and Applied Thermodynamics, Institute of Chemistry for Energy and Environment, University of Cordoba, Campus Rabanales, Ed. Marie Curie, E-14014 Córdoba, Spain; z52chpem@uco.es (M.C.);

**Keywords:** hemeprotein, gold nanoparticles, electron transfer, biosensor, electroactivity

## Abstract

Electrochemical biosensors are widely used in a multitude of applications, such as medical, nutrition, research, among other fields. These sensors have been historically used and have not undergone many changes in terms of the involved electrochemical processes. In this work, we propose a new approach on the immobilization and enhancement of the electrochemical properties of the sensing layers through the control and bioconjugation of hemoproteins (hemoglobin, myoglobin, and cytochrome C) on anisotropic gold nanoparticles (gold nanotriangles (AuNTs)). The hemeproteins and the AuNTs are mixed in a solution, resulting in stable bioconjugates that are deposited onto the electrode surface to obtain the biosensors. All the systems proposed herein exhibited direct well-defined redox responses, highlighting the key role of the AuNTs acting as mediators of such electron transfers. Several protein layers surrounding the AuNTs are electroactive, as demonstrated from the charge measured by cyclic voltammetry. The retention of the stability of the hemeproteins once they are part of the bioconjugates is evidenced towards the electrocatalytic reduction of hydrogen peroxide, oxygen, and nitrite. The parameters obtained for the proposed biosensors are similar or even lower than those previously reported for similar systems based on nanomaterials, and they exhibit attractive properties that make them potential candidates for the latest developments in the field of sensing devices.

## 1. Introduction

Most of the commercial and non-commercial biosensor devices used are based on electrochemical enzymatic sensors that perform indirect measurements based on the quantification of species such as oxygen (O_2_) and hydrogen peroxide (H_2_O_2_) [1]. These sensors are classified as enzymatic biosensors of first-generation. The first enzymatic sensor was developed by Clark et al. [2]. In this pioneering work, they developed a sensor based on the indirect measurement of the O_2_ generated as a consequence of the selective reaction of glucose with the enzyme glucose oxidase. However, it presented several limitations that needed to be addressed, such as its dependence on O_2_ or the interference of electroactive species [3,4]. Since its apparition, a multitude of approaches have been described for the determination of species with high importance and relevance in medicine and in nutrition, such as glucose [5,6], vitamin C [7,8], and lactate [9,10], among others. In the case of second-generation enzyme sensors, the electrochemical response occurs because of electron transfer mediated by the use of electroactive species with the active center of the enzyme. This fact eliminates the dependence on O_2_ and H_2_O_2_ for this type of sensors, and also allows the decrease of the applied potential, avoiding interferences, although it presents problems in terms of stability, and therefore, possible leaching of the mediator, losing activity of the sensor. These problems of leaching and therefore loss of activity can be solved by the development of third generation sensors, where there is a direct electronic transfer (DET) from the electrode surface to the active site of the enzyme, so it is of special interest regarding its study and optimization [11]. On the other hand, it is worth highlighting the great progress that is being made in the development of non-enzymatic sensors. These sensors are based on the direct detection of the analyte through the use of composites and nanomaterials related to the analyte of interest, as has been observed for molecules such as H_2_O_2_, sulphites, and nitrites [12,13,14,15]. These sensors have some advantages over enzymatic sensors—such as the lack of need for biological compounds that can degrade and therefore lead to a loss of sensor performance—but also have a number of limitations—such as the passivation of its surface by poisoning with other materials, or the low specificity with the analyte of interest that instead offers the enzymatic sensors to their analytes.

Over the years, new methodologies have been developed to improve the sensing properties of electrodes with the introduction of electroactive species, such as mediators [16] and nanoparticles [17,18], for the optimization and development of first-, second-, and third-generation sensors. The latter are particularly interesting and, within the wide variety of systems that are reported, those based on gold nanoparticles (AuNPs) stand out [19]. This can be easily explained by considering the excellent biocompatibility of AuNPs [20], among their attractive surface chemistry and optical properties [21,22,23].

In this regard, the evaluation of the direct electrochemistry that proteins and enzymes present with when they are immobilized at electrodes can be used as a starting point for building electrochemical instrumentation, such as biosensors, bioreactors, and even medical devices [24]. Furthermore, these studies can also establish models to accurately understand the electron transfer process in real biological systems. As mentioned above, to achieve this, it is essential to select a suitable film or support for the electrode surface. In a previous work, we have described the direct electrochemistry and electrocatalysis towards H_2_O_2_ and O_2_ of bioconjugates formed by the electrostatic interaction of Hb immobilized on spherical AuNPs, deposited on a glassy carbon electrode [25]. The results obtained revealed that the mode of binding of the hemeprotein on the AuNP surface does not affect its structural integrity, and we have taken them as a starting point for a more advanced design, as is the one proposed in this paper. More recently, Niu et al. have proposed a new third generation biosensor based on the use of triangular gold nanoparticles (AuNTs) together with horseradish peroxidase (HRP) [19]. The use of AuNTs constitutes a kind of nanomaterial that presents important improvements in the electrochemical signals, mainly due to the presence of hot spots in the vertices of the nanostructures, as it has been demonstrated by their extensive use in photothermal and theragnostic applications, that are mainly based on the Raman enhancement and the near infrared absorption properties. In this system, the presence of AuNTs produces an improvement of the electron transfer rate constant with respect to the direct immobilization of HRP on the electrode thanks to its physicochemical properties, such as large surface area and high conductivity, as already observed in the development of other biosensors based on AuNTs [26,27,28,29].

The design proposed herein is prepared through a new protocol for the design of the sensing layer of future electrodes, favoring their optimization, and leading to the improvement of their electrical properties. For this purpose, different hemeproteins have been used to determine the different electron transfer constants established with AuNTs. Specifically, we have selected proteins that are key to the O_2_ transport, such as Hemoglobin (Hb) and Myoglobin (Mb) [30], in addition to Cytochrome c (Cyt c), which is involved in the electron transport chain of mitochondria [31]. The confirmation of the functionality of the bioconjugated hemeproteins was addressed by checking their redox response once deposited on a glassy carbon electrode, and by evaluating the electrocatalytic response towards the reduction of H_2_O_2_, O_2_, and nitrite ions. We have selected these analytes considering their importance for both analytical and synthetic purposes, in natural and artificial systems [32,33,34]. 

## 2. Experimental Section

### 2.1. Chemicals

Hemoglobin from bovine blood (Hb), Myoglobin (Mb), and Cytochrome C (Cyt c) were purchased from Sigma-Aldrich and used without further purification. Hydrogen tetrachloroaurate trihydrate (from 99.99% pure gold), sodium thiosulfate hexahydrate, and all the supplementary chemicals were of analytical grade, and solutions were prepared with 18.2 MΩ deionized water by Millipore system.

### 2.2. Characterization Techniques

The TEM images were obtained with a JEOL JEM 1400 instrument operating at 80–120 kV, and analyzed using Image Pro Plus software (Servicio Apoyo a la Investigacion (SCAI) Universidad de Córdoba). Samples were prepared onto formvar-coated Cu grids (400 mesh, Electron Microscopy Sciences). The grids were immersed in an AuNT diluted solution for at least one hour and after that time, then dipped in water for an extra hour to ensure the removal of unfixed particles, and finally dried at room temperature. 

The absorbance spectra between 250 and 1600 nm (fixed 1 nm bandwidth) were recorded using a Jasco V-670 UV-vis-NIR spectrophotometer using a UV quartz cell of 2 mm path length.

Electrochemical experiments were performed using an Autolab (Ecochemie model Pgstat30) instrument controlled by GPES 4.9 software for the recording of the experiments and data acquisition. A standard three-electrode cell comprising of a platinum coil as the counter electrode, a 3 M Ag/AgCl electrode as the reference electrode, and a glassy carbon (GC) as the working electrode (BASi Research Products, 3.0 mm diameter) were used. All measures concerning bioconjugates were recorded in 10 mM phosphate buffer solution at pH 7.4, unless otherwise stated. Before each experiment, the GC electrode was polished with 0.3 μm alumina on a microcloth and subsequently sonicated in an ultrasound bath. For the different measurements, the dissolved oxygen was removed by bubbling and maintaining an N_2_ atmosphere. Finally, the clean electrode was cycled in a 50 mM phosphate buffer solution at pH 7.4 until a stationary voltammogram (potential range between −1.1 and 1.1 V)—characteristic of a clean GC electrode—was obtained. 

### 2.3. Synthesis of AuNTs

**Scheme 1:**
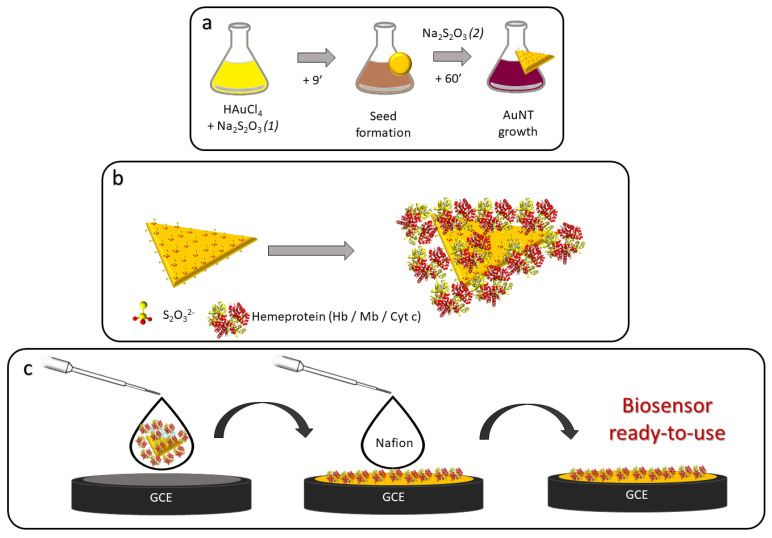
Schematic representation of (**a**) the synthesis procedure for preparation of AuNT using a seed-mediated protocol, (**b**) immobilization of the hemeproteins onto AuNT, and (**c**) modification of the solid GCE by drop casting employing concentrated bioconjugate solutions.

The synthesis of AuNTs was made by following the seed-mediated growth method, described and optimized by several research groups (Scheme 1a) [35,36]. Briefly, we obtain the nanoparticles from the reduction of HAuCl_4_ by Na_2_S_2_O_3_ in an aqueous media. On a first step, the seed solution is prepared by mixing 30 mL of 0.5 mM Na_2_S_2_O_3_ with 25 mL of 2 mM HAuCl_4_ in a 100 mL Erlenmeyer flask. The previous solution is kept under stirring for 9 min and, during this time, it changes its color from an initial pale yellow, to light brown, dark brown, and, finally, deep red. After that time, a second addition of 12.5 mL of 0.5 mM Na_2_S_2_O_3_ promotes the growth of the seeds, and thus, the formation of the AuNTs. The previous mixture is kept under stirring for 45 min and, as a result, we obtain a colloidal suspension of AuNTs of different sizes, which should be capped with thiosulphate anions. The evolution of the reaction was monitored by UV-vis-NIR spectroscopy and, at the end of the process, the LSPR band presented a maximum absorbance centered at ca. 970 nm, confirming the effectiveness of the synthesis (Figure S1a). TEM micrographs of the obtained AuNTs allow us to confirm that our nanoparticles present an average side length of 80 nm (Figure S1b). 

### 2.4. Formation of the Colloidal Suspension of Bioconjugates

To ensure the stability of the colloidal suspension, the concentration of AuNTs was adjusted to 0.07 nM in the presence of 10 μM protein (Hb, Mb, or Cyt c) in an aqueous solution (Scheme 1b). The immobilization of the protein around the gold core is mainly taking place by electrostatic interactions giving place to a protein corona that is composed of several protein layers. Once prepared, the samples were incubated at 4 °C for at least 30 min. Prior to use, they were centrifugated to obtain concentrated samples that ensure the acquisition of good quality electrochemical signals. Through this methodology, the optimization of the protein concentration immobilized on the gold nanoparticles is carried out in the solution before its immobilization on the electrode. This optimization has been taken into account for the electrochemical experiments carried out, saving in the amount of protein used and avoiding the interference and unspecific protein adsorption on the clean electrode that occurs with other methodologies used, such as the direct drop casting of the proteins on the electrodes already modified with nanomaterials. 

### 2.5. Preparation of the Biosensor Platforms

Freshly prepared and concentrated bioconjugates were deposited by drop casting on the surface of a clean GC electrode (3 μL/sample) and were left to dry at room temperature (Scheme 1c). The high solubility of the bioconjugates recommended the use of a thin film of Nafion (0.1% ethanolic solution) to ensure integrity of the platform during the experimental measurements, and thus the electrochemical response does not disappear for the long time experiments. This thin Nafion layer shows no electrochemical signal in the measurements.

## 3. Results and Discussion

### 3.1. Electrochemical Behavior of Hemeproteins Immobilized on AuNT

**Figure 1:**
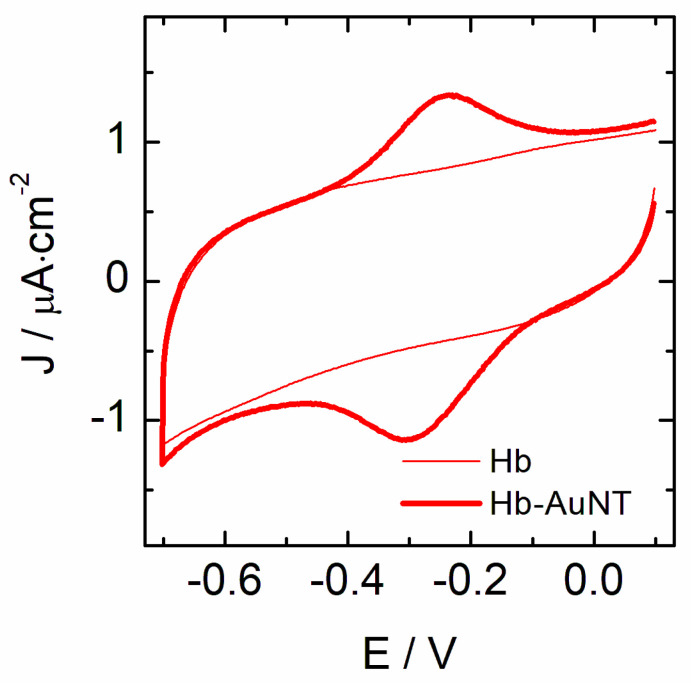
Cyclic voltammograms of Hb and Hb-AuNTs drop casted on a GC electrode in a 10 mM phosphate buffer at pH 7.4 (scan rate 0.1 V/s).

The presence of heme iron porphyrin in the structure of some proteins makes direct electron transfer possible through the heme Fe(III)/Fe(II) species. However, this reaction is slow due to the location of the electroactive group buried in the nonconductive peptide chains. The enhancement of this electron transfer process can be achieved by modifying the electrode surface [37,38] or by using composite materials that include the protein together with some conductive elements, as is the case of the gold nanoparticles forming nanobioconjugates [25]. In the present case, the strategy followed had been the preparation of the bioconjugates AuNT-protein that, upon stabilization, were deposited on a GC electrode surface by a drop casting methodology for electrochemical evaluation. Then, nanobioconjugates formed by AuNTs, with either Hb, Mb, or Cyt c, were checked for electron transfer capability. As it is shown in Figure 1, the presence of Hb-AuNTs bioconjugates on the electrode surface brings about the appearance of the Fe(III)/Fe(II) redox signal at −0.300 and −0.237 V, in contrast to the behavior observed when only Hb is deposited on the surface that shows no peak in the potential interval where the redox pair is currently observed.

**Figure 2:**
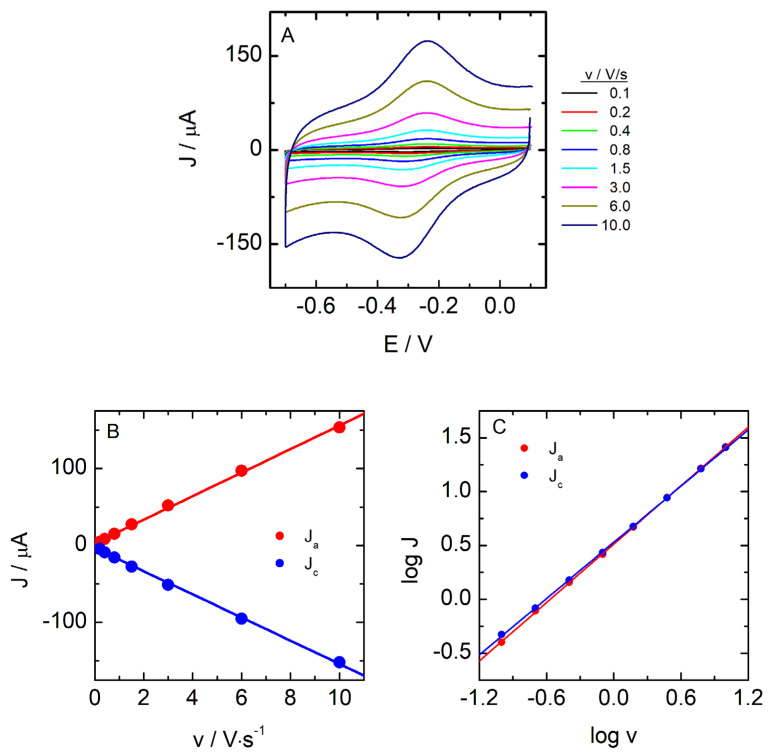
(**A**) Cyclic voltammograms of Hb-AuNT nanobioconjugates drop casted on a GC electrode at different scan rates in a 50 mM phosphate buffer at pH 7.4, (**B**) linear variation of the current against the scan rate, and (**C**) logarithmic plots for the anodic and cathodic currents.

The cathodic and anodic peaks observed for Hb-AuNTs show a peak separation of 63 mV and an E°’ value of −0.268 V. When the cyclic voltammograms are recorded as a function of the scan rate, increases of the current density and peak potential separation are obtained (Figure 2A). The current increase with the scan rate shows a linear variation, indicating that the process is taking place on the surface, as is also evidenced by the logarithmic plot that gives slopes close to unity according to Equation (1) (Figure 2B,C).

(1) $$I_{p}=\frac{n^{2}F^{2}}{4RT}\cdot v\cdot A\cdot \Gamma =\frac{nFQv}{4RT}$$

**Figure 3:**
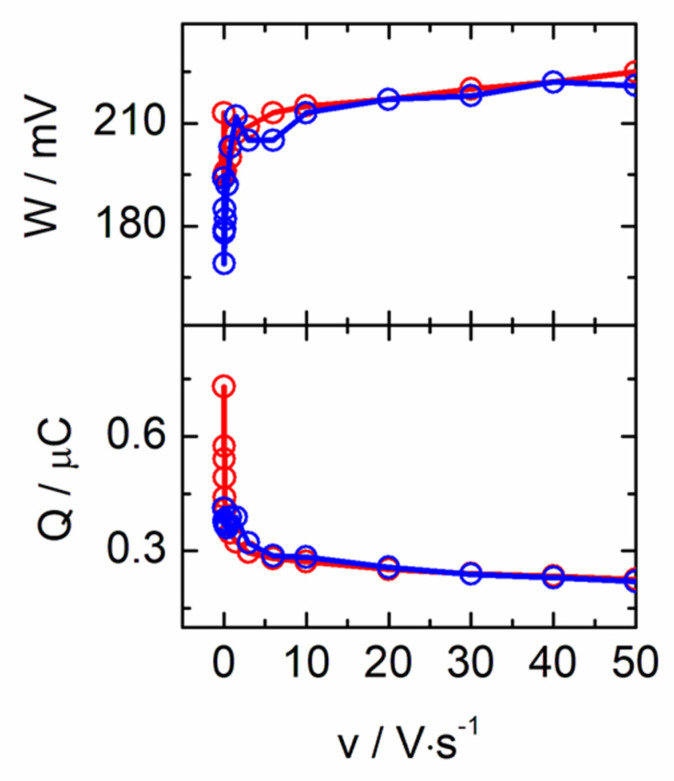
Charge density (Q) and peak half width (W) of the Hb-AuNTs deposited on a GC electrode in a 50 mM phosphate buffer at pH 7.4, as a function of the scan rate.

In these systems, the charge involved in the electron transfer process can be determined from the integration of the voltammetric peaks upon background subtraction, and the results are plotted in Figure 3 as a function of scan rate, along with the values of the peak half width, W. The shape of the cathodic and anodic peaks of the voltammograms are nearly symmetric but present a half width higher than the theoretical value for an ideal process (90.6/n mV). This fact can be explained either as a consequence of the spatial distribution of redox centers in multilayers surrounding the nanoparticle [39], or as a consequence of a heterogeneous population of proteins adsorbed in a non-uniform manner that gives rise to different adsorption environments and hence a distribution of different redox states [40,41,42]. The W parameter presents a smaller value at low scan rates and suddenly increases, achieving a constant value at scan rates higher than 5 V/s. A parallel comportment to W can be observed in the charge involved in the anodic and cathodic processes that decrease to reach a constant value. This behavior has been seen in other systems and has been explained as due to the presence of protein multilayers adsorbed on the surface of the electrode and that, only at low scan rates, they can undergo the redox process efficiently. However, when the scan rate increases, the charge density decreases until it reaches a constant value, evidencing that the electron exchange only occurs with the protein layers closest to the electrode surface [42]. Based on the above, an analysis of the situation can be made, considering the Equation (2), which allows determining the surface coverage (Γ) if the charge involved (Q) and surface area (A) are known.

(2) Q=n·F·A·Γ

The surface coverage determined at the lower scan rates is 2.17 × 10^−10^ mol/cm^2^, and decreases up to 7.02 × 10^−11^ mol/cm^2^ at scan rates where the amount of charge becomes constant. Comparing to the theoretical monolayer coverage (5.18 × 10^−12^ mol/cm^2^) for Hb and, considering that each unit is composed of 4 monomers with its corresponding heme group that could exchange 4 electrons, the amount of electroactive protein molecules is much greater than the corresponding to a monolayer, in particular, at the low scan rate regime. When analyzing the result at higher scan rates, the charge density corresponds to the monitorization of two to three layers of protein around the AuNT—that is, only the first two protein layers are involved in the electronic exchange. 

The time scale required to change the oxidation state of a redox species confined to the surface comprises of two complementary mechanisms. On the one hand, the charge transfer mechanism involves the direct electron transfer between the electrode and the redox site located at a distance at which, probably, the tunnel effect must act. On the other hand, the spread of redox reactions within the film and beyond the tunnel distance from the electrode requires the exchange of electrons between neighboring sites. This process, known as charge transport, is diffusive in nature and is assisted by segmental movements of the polymeric film, making it easier for neighboring redox sites to approach each other long enough for an electron hopping event to occur [43]. The transport and charge transfer rates and their dependence on the experimental variables have been studied, and it has been found that the results obtained in randomly arranged films cannot be extrapolated to the organized multilayers cases that exhibit the ability to control the nature and the charge of the layers. The model of Laviron [39,44] deals with redox centers located in well-defined planes, while the real cases have a less homogeneous distribution. A more recent model [43] allows the potential to vary with the distance from the redox center to the electrode surface. In this case, the potential difference between the oxidation and reduction peaks (ΔE) at low scan rates increases as the apparent diffusion coefficient decreases. This effect is associated with limitations in charge transport. In this case, ΔE reaches a constant value at about 58 mV, very close to the value expected for a diffusion-controlled system. The Laviron model that considers redox sites located in planes parallel to the electrode predicts a decrease in this value when there is an increase in v, but this occurs when the diffusion can penetrate only the first plane and, therefore, the thin-film behavior is restored. When the charge transfer is slower than the charge transport, the first process masks the separation of peaks and does not allow observing the diffusive phenomenon.

**Figure 4:**
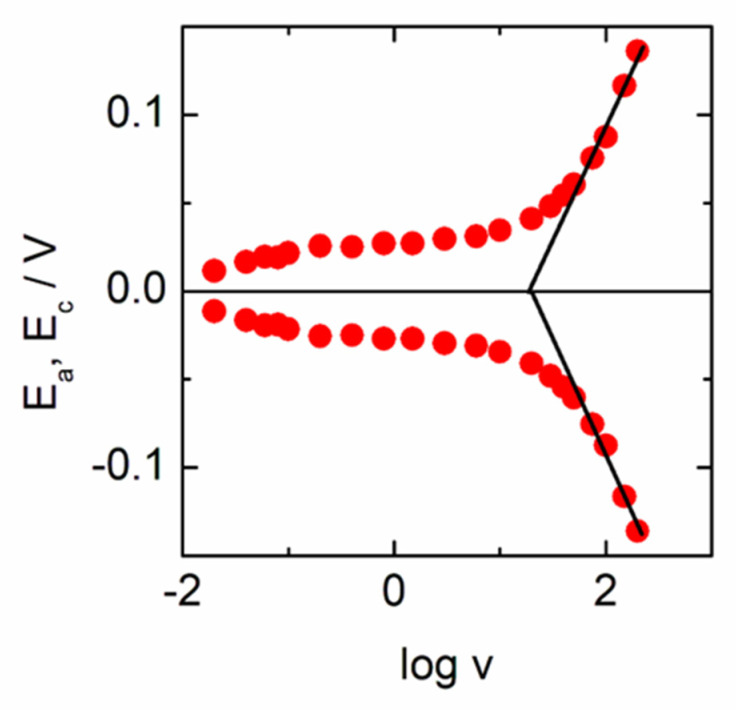
Plot of the cathodic and anodic peak potentials of the Hb-AuNTs as a function of the scan rate. Results are obtained from the voltammograms of Figure 2A.

As seen in Figure 4, the peak potentials for the Hb-AuNTs shows a small separation of peaks at a low scan rate, which increases slightly up to the change, becoming more important at very high scan rates showing two branches that seem to retain some symmetry with respect to the values of the average potentials (Figure 4). Under these conditions, it is possible to determine both the values of α and k_s_ based on the Laviron method [39,44]. At high speeds, the values of E_p_ vary linearly with log v and, since these branches reach values of ΔE > 200/n mV, the Equations (3)–(5) can be used to determine the α and k_s_ parameters.

(3) $$E_{p}=E^{o’}+\frac{2.303RT}{\alpha nF}\left(\frac{log\alpha nF}{RT-logk_{s}}\right)+\frac{2.303RT}{\alpha nFlogv}$$

(4) $$logk_{s}=\alpha \times \log\left(1-\alpha \right)+\left(1-\alpha \right)\times log\alpha -log\frac{RT}{nFv}-\frac{\alpha \cdot \left(1-\alpha \right)nF\eta }{2.3RT}$$

(5) $$k_{s}=\frac{\alpha \cdot n\cdot F\cdot v}{RT}$$

In the present case, values for α and k_s_ of 0.5 and 138 s^−1^, respectively, have been obtained. The k_s_ value is much greater than those found for this same protein forming parts of different composite materials, both nanometric and polymeric, or with nanotubes or carbon aerogels [45], Fe_3_O_4_ nanostructured materials [46], gold nanorods modified with silica [47], gold nanoparticle assembled capsules [48], multiwalled carbon nanotubes-zinc oxide composites [49], gold nanoshells [50], and nickel oxide nanoparticles [51].

The results obtained for the Mb- and Cyt c-AuNTs are similar to these described for the Hb-AuNTs system, also showing voltammogram shapes that are typical for redox processes in the adsorbed state. The parameters obtained for the studied systems are gathered in Table 1. 

These data show that the presence of the AuNTs facilitates the fast electron transfer of the heme iron groups in the three proteins studied. It is interesting to note that although both Hb and Mb possess a 5c heme b as a prosthetic group with a His as the proximal ligand, and Cyt c has a different one, the three nanobioconjugates behave similarly. In the case of Cyt c, the native protein possesses a heme c with a 6c conformation that involves the axial coordination of Met 80 and His 18 that are responsible for the relatively high redox potential that allows the protein to exert its functional role in the respiratory chain [24]. It has been reported that the coordination of the Fe ion changes upon interaction with some nanomaterials, changing the coordination of Met 80 by a His residue that gives place to a bis-His complex that shifts the redox potential negatively and induces a structure that presents peroxidase activity [52]. 

### 3.2. Electrocatalysis of the Nanobioconjugates

**Figure 5:**
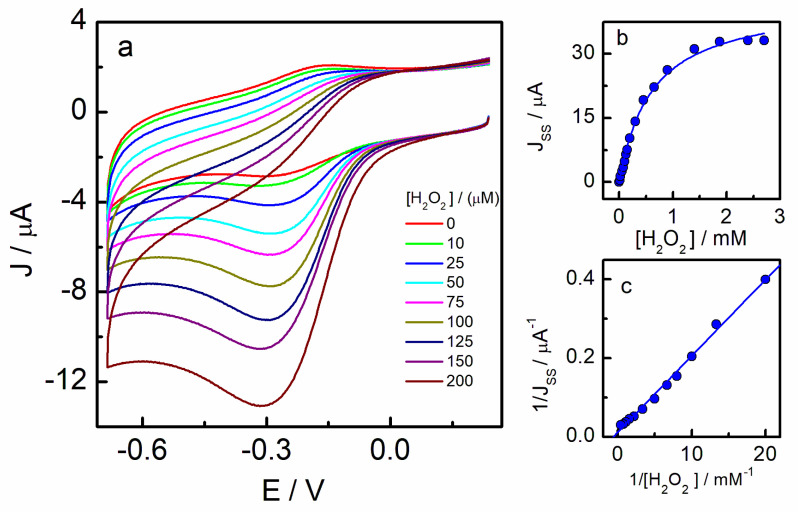
Electrocatalytic response of the Hb-AuNT-GC system for H_2_O_2_ detection. (**a**) Cyclic voltammograms recorded after multiple additions of H_2_O_2_ in a 10 mM phosphate buffer solution at pH 7.4, (**b**) plot of the current intensity measured at the potential maximum as a function of H_2_O_2_ concentration, and (**c**) Lineweaver–Burk plot.

Taking into account the redox response of the nanobioconjugates, it seems interesting to check their capacity as sensor systems against some molecules, such as H_2_O_2_ [53,54,55], NaNO_2_ [56,57], and O_2_ [57,58], that have been shown to be catalyzed by these proteins under determined conditions. Figure 5 shows the voltammograms of the Hb-AuNT deposited on a GC electrode obtained in solutions with increasing amounts of H_2_O_2_ in a phosphate buffer at pH 7.4. It is observed that the reduction current gradually increases, while the oxidation rate decreases until finally disappears. This behavior is typical of electrocatalytic systems, in which the reduced state of Hb that is generated is immediately oxidized with the H_2_O_2_ present in solution. While in the absence of H_2_O_2_, the voltammogram presents characteristic redox peaks of Hb at E°’ of −0.268 V; the addition of hydrogen peroxide results in a current increase of the Hb-Fe(III) reduction peak, concomitant with the decrease; and finally the disappearance of the Hb-Fe(II) oxidation peak intensity. Such behavior indicates that this platform functions as a biosensor and shows excellent electrocatalytic activity towards H_2_O_2_ reduction [59]. The cathodic current (measured at a constant potential of −0.30 V) increases linearly with the analyte concentration until c.a. 500 μM. Up to this value, a constant intensity current is assessed—that is, reaches a plateau—characteristic of the Michaelis–Menten mechanism. The apparent Michaelis–Menten constant (K_M_^app^) is a reflection of the analyte-substrate kinetics, and it can be obtained from the electrochemical version of the Lineweaver–Burk equation (Equation (6)),

(6) $$\frac{1}{J_{ss}}=\frac{1}{J_{max}}+\frac{K_{M}^{app}}{J_{max}\cdot C}$$

where J_ss_ is the steady-state current after the addition of the substrate, C is the substrate bulk concentration, and J_max_ is the maximum current measured under saturated substrate conditions. Thus, K_M_^app^ values are obtained from the analysis of the slope and the intercept for the plot of the reciprocal of the steady state current versus the reciprocal concentration of the analyte. The effectiveness of the proposed biosensors, as evidenced by the calculated K_M_^app^ values (summarized in Table 2), are comparable to or lower than those previously reported in the literature for similar systems that present good response for the electrocatalysis of H_2_O_2_ and that involve the use of hemeproteins: Hb-cAuNP/GCE (1.2 mM) [21], Mb-AuNPs-CNs/GCE (0.3 mM) [60], and Cyt c/AuNP/carbon paste (2.3 mM) [61] within others [24,62]. In addition, it is noteworthy that the values presented in this work were better than those reported for nanozymes based on hemeprotein active sites for the detection of H_2_O_2_ in living cells [63]. Thus, the obtained results indicated that the biosensors maintain a higher biological affinity to H_2_O_2_ because the hemeproteins retain their catalytic activity.

The nitrite reductase activity of the Fe-heme group of these proteins upon bioconjugate formation has also been examined. One of the most accepted mechanisms for the heme iron catalytic nitrite reduction [62,64,65,66] consists of the disproportionation reaction of the HNO_2_ species that occurs in acid media, giving place to NO and NO_3_^−^ anions (reactions (i) and (ii)). The NO species interact with the heme-Fe(II) form of the protein (reaction (iii)), forming the ferrous nitrosyl complex (reaction (iv)) that reduces at the electrode releasing heme-Fe(II) that continues the catalytic cycle (reaction (v)). The later reaction occurs at around −0.71 V.

(i) $$NO_{2}^{-}+H^{+}⇄HNO_{2}$$

(ii) $$3HNO_{2}⟶2NO+NO_{3}^{-}+H^{+}+H_{2}O$$

(iii) $$Heme-Fe\left(III\right)+H^{+}+e^{-}⇄Heme-Fe(II)$$

(iv) $$NO+Heme-Fe\left(II\right)⟶Heme-Fe\left(II\right)-NO$$

(v) $$Heme-Fe\left(II\right)-NO+H^{+}+e^{-}⟶Heme-Fe\left(II\right)+product+H_{2}O$$

**Figure 6:**
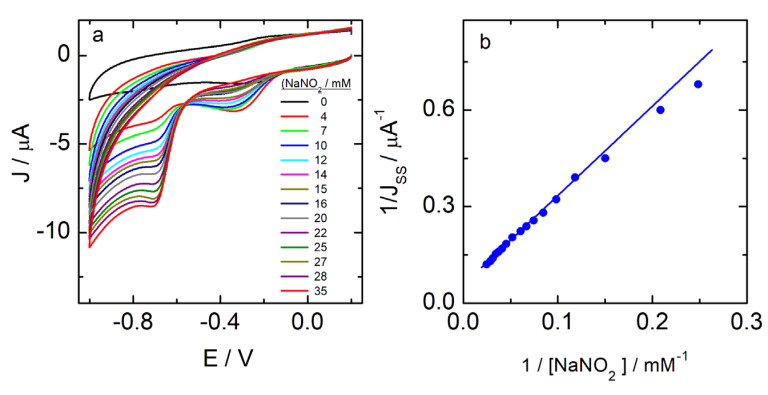
(**a**) Cyclic voltammograms for the electrocatalytic response of the Hb-AuNTs system for nitrite ions detection. The voltammograms were recorded after multiple standard additions of NaNO_2_ solution to a 10 mM phosphate buffer solution at pH 5; (**b**) Lineweaver–Burk plot.

However, there have been many works related to this topic as the obtained voltammetric behavior is very dependent on the experimental conditions and the nature of the enzyme used to build the biosensor. In the present systems, a reduction peak at −0.71 V appeared in the presence of nitrite concentrations, which increased with an increasing concentration from 1 mM up to 50 mM NaNO_2_, where it reached saturation (Figure 6). The electrochemical reduction of nitrite ions is a very complex process, and an important pH influence was also observed. In this sense, the peak at −0.3 V obtained in the present case, is not always present. We observed the peak at −0.3 V only at low nitrite concentrations and, at higher concentrations, this peak moved to negative values in parallel with the appearance of the peak at −0.71 V, to only the latter was observed. It can be speculated that there are two different regimes for nitrite reduction, and these take place through the Fe(II) and Fe(I) states of the protein. Under these conditions, in our system, we could see both routes, catalysis through Fe(II) at −0.3 V and through Fe(I) at −0.71 V, the latter being the predominant under most of the experimental conditions. 

The observed behavior is similar for the three protein bioconjugates, indicating that it is dictated by the used nanomaterials and the type of interactions, and less dependent on the nature of the protein. We have also analyzed the systems by following Michaelis–Menten formalism and the K_M_^app^ values obtained are gathered in Table 2. The best performance of the Cyt c-AuNTs could be ascribed to the smaller size of the protein and the change in the heme conformation as described above.

**Figure 7:**
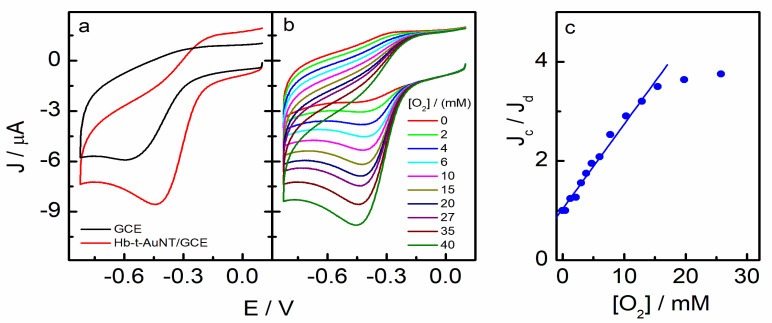
Electrocatalytic response of the Hb-t-AuNTs system for O_2_ detection. (**a**) Cyclic voltammograms for the reduction of O_2_ at equilibrium concentration recorded by using a bare GCE (black line), and one of the biosensors described in this work (red line) in a 10 mM phosphate buffer solution at pH 7.4; (**b**) Effect of the addition of increasing concentrations of O_2_; (**c**) Catalytic efficiency as a function of O_2_ concentration.

Finally, we have checked the ability of these platforms against the reduction of O_2_. The presence of O_2_ molecules in the solution provoked a pronounced increase in the reduction current (Figure 7a, red line) in respect to the signal obtained with the bare GC electrode. Moreover, a displacement of the reduction signal of around 0.2 V is obtained that should be ascribed to the electrocatalytic reaction [67]. The reaction involved can be expressed as follows:

O_2_ + 2 heme-Fe(II) + 4H^+^ + 2e^−^ ↔ 2 heme-Fe(III) + 2H_2_O

Besides the mentioned decrease in the overpotential necessary to observe the O_2_ electrocatalysis, we have also studied the sensitivity to its detection (Figure 7b). To get these data, we first eliminated the oxygen present at the equilibrium concentration in the buffer solution by bubbling N_2_. Then, a controlled amount of air was introduced within the cell through a syringe and the concentration was determined by considering the air composition (21% O_2_).

The catalytic efficiency is expressed as the ratio of the reduction peak current in the presence (J_c_) and absence of oxygen (J_d_). We have plotted the ratio J_c_/J_d_ against the O_2_ concentrations monitored, and a linear behavior had been obtained with slopes of 79, 88 and 170 M^−1^ for Cyt c-, Mb-, and Hb-AuNTs bioconjugates, respectively. These values can be correlated with the sensitivity of these platforms to O_2_ electrocatalysis.

Overall, the three platforms studied in this work constitute good systems for the sensing of H_2_O_2_, nitrite ions, and molecular O_2_. As far as we know, the mediation of the AuNTs to sensing analytes through heme-proteins have only been applied with horseradish peroxidase by a different approach of constructing an AuNTs/CILE electrode, with very good results for trichloroacetic acid and nitrite [19]. These results, together with other findings where these nanomaterials have been proven to be effective in high signal improvement for sensing [26,27,28], open new avenues for their use in biosensors of high performance. 

Although this work presents a different methodology to the one used for the generation of new, more efficient and sensitive biosensors, and therefore we do not emphasize in the repeatability, stability, and reproducibility of the platforms, we must mention that thanks to the preparation in the first instance of the bioconjugate and its subsequent deposition on the electrode, a greater reproducibility is achieved, as well as a greater stability of the protein against factors such as pH and temperature, as we have been able to prove in previous studies [25]. 

## 4. Conclusions

Herein, we have demonstrated how the configuration and formation of stable bioconjugates of hemoproteins with AuNTs can increase the sensitivity of biosensors by increasing their electron transfer rate constant. The determination of H_2_O_2_, nitrite ions, and molecular O_2_ based on the catalytic behavior that different hemeproteins (Hb, Mb, and Cyt c) present when they were conjugated to AuNTs and deposited onto GC surfaces was tested. All the proposed systems present a couple of peaks characteristic of a redox quasi-reversible process through a direct electron transfer through the AuNTs with the surface of the electrode, and based on our calculations, the registered current is caused by at least two protein layers surrounding the AuNTs, meaning that only such layers can exchange electrons effectively enough to participle in the electron transfer process.

In comparison with previous studies, the electron transfer constants obtained are higher, indicating that the electron transport is favored. This different behavior observed may be related to the optimization of the conformation of the protein layers surrounding the complete surface of the AuNTs, in comparison to the excess of proteins on the nanomaterial previously deposited on the surface as is made in the previously reported cases. This improved immobilization of the proteins on the surface of the AuNTs, and their subsequent deposition on the electrode, also enhances the analytical properties for the detection of H_2_O_2_, NaNO_2_, and O_2_. This proof of concept can be applied for the prior modification of nanoparticles with stabilizing proteins or enzymes for the future development of enzymatic biosensors of first-, second-, and third-generations and its application to commercial devices such as a glucometer or continuous system. Besides, the response of the biosensor to nitrite detection is also remarkable, considering the significantly smaller number of devices capable of detecting this analyte.

## Figures and Tables

**Table 1:** Electrochemical parameters obtained from the analysis of the bioconjugates.

Biconjugate	E_c_/V	E_a_/V	E°’/V	ΔE/mV	k_s_/s^−1^
**Hb-AuNT**	−0.300	−0.237	−0.268	63	138.0
**Mb-AuNT**	−0.280	−0.237	−0.258	43	104.1
**Cyt c-AuNT**	−0.290	−0.252	−0.271	38	148.4

**Table 2:** Electrocatalytic parameters determined for H_2_O_2_ and NO_2_^−^ analytes in the presence of heme-based bioconjugates dropped in a GC electrode.

	Platform	Hb-AuNT	Mb-AuNT	Cyt c-AuNT
H_2_O_2_	K_M_^app^/mM	0.808	0.602	0.195
	J_max_/μA	50.2	38.0	15.5
	S/μA·mM^−1^	48.7	45.6	45.4
	LOD/µM	18	27	39
NO_2_^−^	K_M_^app^/mM	49	5.3	2.9
	J_max_/μA	17.8	7.7	3.2
	S/μA·mM^−1^	0.2	0.3	0.3
	LOD/mM	1.7	0.8	1.2

## Data Availability

Not applicable.

## Supplementary Materials

The following supporting information can be downloaded at: https://www.mdpi.com/article/10.3390/bios13040467/s1, Figure S1: (a) UV–visible-NIR spectra recorded at the end of the t-AuNT synthesis procedure. (b) TEM image of the same sample before the formation of the bioconjugate.
