# Supplementary material for: Behind the Optimization of the Sensor Film: Bioconjugation of Triangular Gold Nanoparticles with Hemoproteins for Sensitivity Enhancement of Enzymatic Biosensors
Source: Biosensors (Basel). 2023 Apr 10;13(4):467. doi: 10.3390/bios13040467 (PMC10136871; doi:10.3390/bios13040467)
Supplement: Supplementary file 1 [file biosensors-13-00467-s001.zip › biosensors-2279016-supplementary.pdf]

Behind the Optimization of the Sensor Film: Bioconjugation of Triangular Gold Nanoparticles with Hemoproteins for Sensitivity Enhancement of Enzymatic Biosensors

Miriam Chávez, Ángela Fernandez-Merino, Rafael del Caño, Guadalupe Sánchez-Obrero, Rafael Madueño, Manuel Blázquez and Teresa Pineda *

Department of Physical Chemistry and Applied Thermodynamics, Institute of Chemistry for Energy and Environment, University of Cordoba, Campus Rabanales, Ed. Marie Curie, E-14014 Córdoba, Spain; z52chpem@uco.es (M.C.)
* Correspondence: tpineda@uco.es

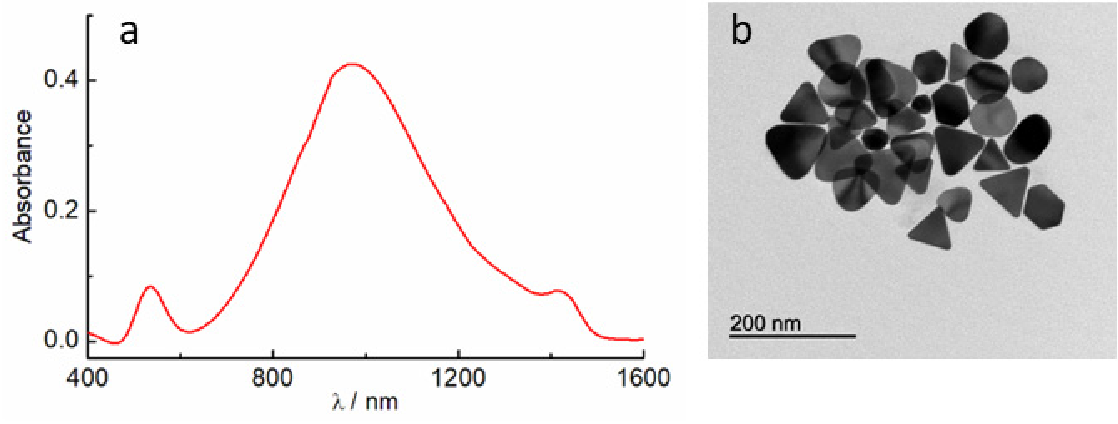

Figure S1. (a) UV–visible-NIR spectra recorded at the end of the t-AuNT synthesis procedure. (b) TEM image of the same sample before the formation of the bioconjugate.
